# Supplementary material for: Identification and Functional Analysis of foxo Genes in Chinese Tongue Sole (Cynoglossus semilaevis)
Source: Int J Mol Sci. 2023 Apr 21;24(8):7625. doi: 10.3390/ijms24087625 (PMC10142177; doi:10.3390/ijms24087625)
Supplement: Supplementary file 1 [file ijms-24-07625-s001.zip › ijms-2287327-supplementary.pdf]

Supplementary Figure S1 The complete ORFs and deduced amino acid sequences of *Csfoxo1a*, *Csfoxo3a*, *Csfoxo3b Csfoxo4 Csfoxo6*and *Csfoxo1a-like*.

Csfoxo1a

```
1        ATGGCGGAAGCGGCGCAGCAGCAGCATCAGCCACACCTGGTGGACATTGACCCGGATTTTGAACCCCTTTCGCGGCCGCGCTCCTGCACT
1         M  A  E  A  A  Q  Q  Q  H  Q  P  H  L  V  D  I  D  P  D  F  E  P  L  S  R  P  R  S  C  T
91       TGGCCCCTGCCCCGGCCGGAGTTCGTTAACCCGGGCGATTCTAACACATCATCACCGGTGCCCTCCGTGAAGCAGGAGCCCGCCGGTAAC
31        W  P  L  P  R  P  E  F  V  N  P  G  D  S  N  T  S  S  P  V  P  S  V  K  Q  E  P  A  G  N
181      CCCGACTTCATCAACAACCTTAGTCTGTTGGAGGAGAGCGAGGACTTGGAGGAGCACAAGCCCGTCCTCCTGTGCGCTGACGGTTTTCAG
61        P  D  F  I  N  N  L  S  L  L  E  E  S  E  D  L  E  E  H  K  P  V  L  L  C  A  D  G  F  Q
271      TGTCAGGAGAACTGCGTCCAGCAGCAACAACCTCTACAGCAGCAACATCAGCAACAACTCCCGCATCACCAGCAGCATCAGCACCATCAC
91        C  Q  E  N  C  V  Q  Q  Q  Q  P  L  Q  Q  Q  H  Q  Q  Q  L  P  H  H  Q  Q  H  Q  H  H  H
361      CACCAGCAGCAGCAGCAGCAGGTGGCTCTTCTCTCCTCACCGGTTGGTTCTTCGTCCTCTGCGGCCGCAGCTGCCGCCGCTGCTGCCCAG
121       H  Q  Q  Q  Q  Q  Q  V  A  L  L  S  S  P  V  G  S  S  S  S  A  A  A  A  A  A  A  A  A  Q
451      AGGAAGAGCAGCTCCTCACGGCGGAACGCGTGGGGGAACATGTCATATGCAGACCTCATCACCAAAGCTATTGAAAGTTCTCCCGAAAAA
151       R  K  S  S  S  S  R  R  N  A  W  G  N  M  S  Y  A  D  L  I  T  K  A  I  E  S  S  P  E  K
541      CGTCTGACTCTGTCCCAGATCTATGACTGGATGGTGAAGAGTGTGCCATATTTCAAGGATAAAGGGGACAGCAACAGCTCTGCAGGATGG
181       R  L  T  L  S  Q  I  Y  D  W  M  V  K  S  V  P  Y  F  K  D  K  G  D  S  N  S  S  A  G  W
631      AAGAACTCCATTAGACACAACCTGTCACTGCACAGCCGCTTTATCCGCGTTCAGAATGAAGGGACGGGAAAAAGCTCCTGGTGGATGTTA
211       K  N  S  I  R  H  N  L  S  L  H  S  R  F  I  R  V  Q  N  E  G  T  G  K  S  S  W  W  M  L
721      AACCCAGAAGGTGGGAAGAGTGGGAAGTCACCACGACGCAGAGCTGCCTCTATGGACAACAACAGCAAGTTCAGCAAAAGCAGAGGAAGA
241       N  P  E  G  G  K  S  G  K  S  P  R  R  R  A  A  S  M  D  N  N  S  K  F  S  K  S  R  G  R
811      GCAGCAAAGAAAAAGCTCTCCCTACAGGGTGGTCCTGACGGAGGTGCTGACAGCCCTGGCTCCTACTCTAAGTGGCCCGGAAGCCCCAAT
271       A  A  K  K  K  L  S  L  Q  G  G  P  D  G  G  A  D  S  P  G  S  Y  S  K  W  P  G  S  P  N
901      TCACACAGCAACGACGACTTTGAGGCATGGAACAGCTTCAGGTCTCGGGCCAGCTCTAATGCTAGCACTCTGAGTGGCCGCCTGTCACCT
301       S  H  S  N  D  D  F  E  A  W  N  S  F  R  S  R  A  S  S  N  A  S  T  L  S  G  R  L  S  P
991      TTTGTGCCTGAGGACGATCTTGGTGAGGGGGAAGTGCATATGGGTTATCCAGGAGCACCTGGAACCAAAATGACATCCACACTGCCAAGT
331       F  V  P  E  D  D  L  G  E  G  E  V  H  M  G  Y  P  G  A  P  G  T  K  M  T  S  T  L  P  S
1081     CTGACAGAGATGACTGGAACTCTGGGACACAGTTCTGAGAATGTGATGGAGAATCTTTTGGACAACCTAAATTTGCTCTCACCCAACAAT
361       L  T  E  M  T  G  T  L  G  H  S  S  E  N  V  M  E  N  L  L  D  N  L  N  L  L  S  P  N  N
1171     TCTCAGGTCGGTGCAGGTGCAACCAGCCCTGGCTCATCCCAGTCGTCCCCGTCTCCTATGATGCAGCCCAGCCCCGGCTACCCTGCTTAC
391       S  Q  V  G  A  G  A  T  S  P  G  S  S  Q  S  S  P  S  P  M  M  Q  P  S  P  G  Y  P  A  Y
1261     AGCTCTGCCAGCATGGCCTCACAGCCTCAACAGGAGTATCGGAAATGTGGGTACGGACAACCTGGAATGAACAGCCTGCCATCTATGCCA
421       S  S  A  S  M  A  S  Q  P  Q  Q  E  Y  R  K  C  G  Y  G  Q  P  G  M  N  S  L  P  S  M  P
1351     CTGCAACCACTGCCAGAGAGCAAGCCCAGTTTTGTATCAGGTATGGGTCAGTACAGCTGCCCAGCTGGGCTGCTGAAGGAGCTGCTAACT
451       L  Q  P  L  P  E  S  K  P  S  F  V  S  G  M  G  Q  Y  S  C  P  A  G  L  L  K  E  L  L  T
1441     TCAGACAGTGACCAGCACGGAGAGCTGATGCCATCAGTGGACACTGTTGTGTCTCAGTCCACAGGAAGCTGCATGCTGCCGCCTTACAGC
481       S  D  S  D  Q  H  G  E  L  M  P  S  V  D  T  V  V  S  Q  S  T  G  S  C  M  L  P  P  Y  S
1531     AGCAGTCAGCATGCACCCAAACTCATGAGTGGAGGGAACGGTCACATGCATGCTCTGTCTCACGGTCACACTCACAGTGTGCACAACCAG
511       S  S  Q  H  A  P  K  L  M  S  G  G  N  G  H  M  H  A  L  S  H  G  H  T  H  S  V  H  N  Q
1621     GCTAAGAATGGCCGTTTGCTAATGTCGCTCCACGGTGGAGGCAGCGCCCATTTGCCTGCAACCAAAAGCCCAATGCAGATGTCATATGGT
541       A  K  N  G  R  L  L  M  S  L  H  G  G  G  S  A  H  L  P  A  T  K  S  P  M  Q  M  S  Y  G
1711     ATATCCAGTCACCACGGAGGGGGAGGCATGCCGGGGGGCTTCTGTCCTCTCAACACAAATGGATATGGTCGACTTGGGCCAATGCTGCCA
571       I  S  S  H  H  G  G  G  G  M  P  G  G  F  C  P  L  N  T  N  G  Y  G  R  L  G  P  M  L  P
1801     TCACACCCAGAGAAGCTCCCCAGCGACTTGGACGACATGTCCATAGAGAAGTTCGAGTTTGACATGGAGACTGTTCTCCATGACACTCTG
601       S  H  P  E  K  L  P  S  D  L  D  D  M  S  I  E  K  F  E  F  D  M  E  T  V  L  H  D  T  L
1891     ATGGATGGGGACTCTCTTGATTTCAACTTTGAACCAATGGTTACACCTCAAGGTTTTACCCACGGGGTTAAGACCACCACACACAGCTGG
631       M  D  G  D  S  L  D  F  N  F  E  P  M  V  T  P  Q  G  F  T  H  G  V  K  T  T  T  H  S  W
1981     GTGTCAGGATAG
661       V  S  G  *
```

Csfoxo3a

```
1        ATGGCCGAGGCGACGAGCGTCGAAAACGAGCCCCCTCTGAAGGTGGAGATCGACCCGGATTTTGAGCCTCAGAAGCGACCACGCTCCTGC
1         M  A  E  A  T  S  V  E  N  E  P  P  L  K  V  E  I  D  P  D  F  E  P  Q  K  R  P  R  S  C
91       ACTTGGCCGCTGCCCCGTCCGGAGTCCGGTGCGGGCAAACCCGGGACCAATGACACTGACGTAATCCCTGAAGAAGATGATGATGAGGAG
31        T  W  P  L  P  R  P  E  S  G  A  G  K  P  G  T  N  D  T  D  V  I  P  E  E  D  D  D  E  E
181      GAGGATGGAGGAGGAGGAGCTGCTCACAAAGCCAGCGCTGGTGTAAGTAAACCGCGGGAGTTGAGCTGCAGCAGCAGCGGGGGCTTCAGC
61        E  D  G  G  G  G  A  A  H  K  A  S  A  G  V  S  K  P  R  E  L  S  C  S  S  S  G  G  F  S
271      TCCGTCTCCCAGCCAGTGGAGGTGCAGCGCGGCCCCCGGAAAGAGGAACTCATCGACGGCTCCCCTTCCTCGGCTCAGCAGACCCCGGCC
91        S  V  S  Q  P  V  E  V  Q  R  G  P  R  K  E  E  L  I  D  G  S  P  S  S  A  Q  Q  T  P  A
361      GCGGCTCTGAGCGGCTCTGCCTCCCAGCAGCTGAGAAAGTCCTCCGCCCGCAGAAACGCCTGGGGCAACTATTCCTATGCAGACCTAATC
121       A  A  L  S  G  S  A  S  Q  Q  L  R  K  S  S  A  R  R  N  A  W  G  N  Y  S  Y  A  D  L  I
451      ACCCAGGCCATCGAGAGCTCCCCGGAGAAACGACTGACCTTGTCCCAGATCTACGACTGGATGGTGAGATCTGTACCGTACTTCAAGGAC
151       T  Q  A  I  E  S  S  P  E  K  R  L  T  L  S  Q  I  Y  D  W  M  V  R  S  V  P  Y  F  K  D
541      AAAGGCGACAGCAACAGCTCTGCAGGCTGGAAGAATTCCATCCGACACAACTTGTCACTGCACAGCCGTTTTGTCAAAGTTCAAAATGAA
181       K  G  D  S  N  S  S  A  G  W  K  N  S  I  R  H  N  L  S  L  H  S  R  F  V  K  V  Q  N  E
631      GGCACAGGGAAAAGTTCCTGGTGGATGGTCAACCCAGAAGGCGGCAAAGGAGGGAAAGCTCCACGTCGCCGGGCCGTATCGATGGATAAC
211       G  T  G  K  S  S  W  W  M  V  N  P  E  G  G  K  G  G  K  A  P  R  R  R  A  V  S  M  D  N
721      AGTAAATACATCAAAGGAGCTCGAGGACGAGCCACCAAGAAAAAAGCCTCACTGCAGGCGGCTCAAGATGGCAGCTCTGAAAGCTCCTCC
241       S  K  Y  I  K  G  A  R  G  R  A  T  K  K  K  A  S  L  Q  A  A  Q  D  G  S  S  E  S  S  S
811      AGCCTCTCAAAATGGACAGGAAGTCCAACCTCCCGAAGTAGTGACGAGCTGGATGCCTGGACGGACTTCCGCTCCAGGACAAATTCTAAT
271       S  L  S  K  W  T  G  S  P  T  S  R  S  S  D  E  L  D  A  W  T  D  F  R  S  R  T  N  S  N
901      GCCAGCACCCTCAGCGGTCGTCTGTCCCCAATCCTGGCCAACCTGGAGCTGGACGAGGTCCCGGATGATGACTCTCCCCTTTCCCCAATG
301       A  S  T  L  S  G  R  L  S  P  I  L  A  N  L  E  L  D  E  V  P  D  D  D  S  P  L  S  P  M
991      CTGTACTCGAGTCCCAGCAGTATGTCTCCGTCCACTGGTCCCACAGGTCTGTCTGACCTGGCAGGTACCATGAACCTTAACGATGGTCTC
331       L  Y  S  S  P  S  S  M  S  P  S  T  G  P  T  G  L  S  D  L  A  G  T  M  N  L  N  D  G  L
1081     TCTGACAACCTGATGGACGACCTTTTAGACAATATCAGCCTGACAGCATCCCAGCAGCCTCCTCCTGGAGAGGAAGACAACGGAGTTAAT
361       S  D  N  L  M  D  D  L  L  D  N  I  S  L  T  A  S  Q  Q  P  P  P  G  E  E  D  N  G  V  N
1171     GGTCAGGGGAGTTCCCTGTTTACCTTCAGCTGCTCAGGAAGCAACCTGGGAAGTCCCTCTGGCAGCTATGGCTCAAACACTCTTTTCAGC
391       G  Q  G  S  S  L  F  T  F  S  C  S  G  S  N  L  G  S  P  S  G  S  Y  G  S  N  T  L  F  S
1261     CCACCTTCCATCACAAGCTTGCGACAGTCACCCATGCAGACCATTCAGGAGAACAAACAGACCACGTTTTCCTGTATCTCACACTTCAGC
421       P  P  S  I  T  S  L  R  Q  S  P  M  Q  T  I  Q  E  N  K  Q  T  T  F  S  C  I  S  H  F  S
1351     GACCACCAGAGTCTGCAAGACATGCTCAGTCTGGACTCCCCCGGCCACAGCAATGTCCTCCTAACCCAGTCTGACCCTCTGATGTCGCAG
451       D  H  Q  S  L  Q  D  M  L  S  L  D  S  P  G  H  S  N  V  L  L  T  Q  S  D  P  L  M  S  Q
1441     GCCAGTACCGCCATTGCTCTGCAAAACTCTCGCCGAAATGCCATGCTGCTGCGCAAAGACCACATGCTGATCAGTGCAGGTCAGGCTCAA
481       A  S  T  A  I  A  L  Q  N  S  R  R  N  A  M  L  L  R  K  D  H  M  L  I  S  A  G  Q  A  Q
1531     AGCTCTTCAGTGCCTGGTTGGCAAGCTGGCTTGTCAACCTCTGACAGCGATGGCGGTCACCGTGAAGCCAAGAGGCCACTCCTGAAGTCA
511       S  S  S  V  P  G  W  Q  A  G  L  S  T  S  D  S  D  G  G  H  R  E  A  K  R  P  L  L  K  S
1621     CCCAGCAAGAATGCAGCCTCTATGCAGCTCAGCTCCAGCCAGGACCGCTTTCCAGCCGATCTGGACCTTGATGTGTTTAATGGCAGCCTG
541       P  S  K  N  A  A  S  M  Q  L  S  S  S  Q  D  R  F  P  A  D  L  D  L  D  V  F  N  G  S  L
1711     GACTGTGACATGGACTCTATCATTCGCAATGAACTGATGGAAGCTGATTGCCTGGACCTCAGCTTCGATTCTTGCCTGACCTCTGCTCAG
571       D  C  D  M  D  S  I  I  R  N  E  L  M  E  A  D  C  L  D  L  S  F  D  S  C  L  T  S  A  Q
1801     AATGCCAACAAGAGTTCAGGAAGCTTCTCGAGCTCAAAACAGACCTCGCCAGAAAGCTGGGTGCCAAGCTGA
601       N  A  N  K  S  S  G  S  F  S  S  S  K  Q  T  S  P  E  S  W  V  P  S  *
```

Csfoxo3b

```
1     ATGGCTCGGGCTGCGCTGGCCGACCCAGTGGCGGACTTGGACGTGGCCATCGACCCGGACTTCGAGCCCCAGAAGCGGCCGAGGTCGTGC
1      M  A  R  A  A  L  A  D  P  V  A  D  L  D  V  A  I  D  P  D  F  E  P  Q  K  R  P  R  S  C
91    ACCTGGCCGCTGCCGCGACCTGAGTCGAGCTCCGCGAAACCGGAGAGCAACGACACCGATATAATCCCCGAGGAGGAGGACGATGAAGAG
31     T  W  P  L  P  R  P  E  S  S  S  A  K  P  E  S  N  D  T  D  I  I  P  E  E  E  D  D  E  E
181   GACGGTGCCACCACCCCTACGGTCGGCATCGACGGCTCCGGTGTGGCGATCGAGGACCAGAGCAGCAACAGCCCCGGCGCTGCGGACGGC
61     D  G  A  T  T  P  T  V  G  I  D  G  S  G  V  A  I  E  D  Q  S  S  N  S  P  G  A  A  D  G
271   TCGCTGTCCTCTCCCGGCCAGGAGAGCGGAGGCTCCCCGCGCTCCTCGCACTCTCCGCCGGCCACCATAGGCGCACTGACTCCCAGCAGC
91     S  L  S  S  P  G  Q  E  S  G  G  S  P  R  S  S  H  S  P  P  A  T  I  G  A  L  T  P  S  S
361   CTGGCCGCTCAGCAGACCCCGAGGAAGGCGTCGTCCCGTCGGAACGCATGGGGGAACCAGTCGTACGCAGACCTGATTACCAAAGCCATC
121    L  A  A  Q  Q  T  P  R  K  A  S  S  R  R  N  A  W  G  N  Q  S  Y  A  D  L  I  T  K  A  I
451   GAGAGCTCACCAGAGCAGAGACTGACACTGTCCCAAATTTACGACTGGATGGTGCGAAACATCCCGTACTTCAAAGACAAAGGCGACAGC
151    E  S  S  P  E  Q  R  L  T  L  S  Q  I  Y  D  W  M  V  R  N  I  P  Y  F  K  D  K  G  D  S
541   AACAGCTCCGCAGGCTGGAAGAACTCCATCCGACACAACCTTTCCCTCCACAGCCGCTTCATTCGAGTCCAAAATGAAGGAACAGGAAAG
181    N  S  S  A  G  W  K  N  S  I  R  H  N  L  S  L  H  S  R  F  I  R  V  Q  N  E  G  T  G  K
631   AGTTCCTGGTGGATGATCAATCCAGAAGGAGGAAAAGGCGGCAAGGCTCCTCGTCGCCGTGCCGTTTCCATGGACAACAGCAACAAGTAC
211    S  S  W  W  M  I  N  P  E  G  G  K  G  G  K  A  P  R  R  R  A  V  S  M  D  N  S  N  K  Y
721   ACCAAGCCCGCCGGTGGCCGTGCGGCAAAAAAGAAGGCGGCGCTGCAAGCCGCAGCCGCTGCAGCTGGGGAAGGTGGAGGAGACAGTCCC
241    T  K  P  A  G  G  R  A  A  K  K  K  A  A  L  Q  A  A  A  A  A  A  G  E  G  G  G  D  S  P
811   TCAGGTCTTTCTAAGTGGCCAGGCAGCCCAACGACCCGCAGCACCGAGGACCTGGATGTTTGGACAGACTTCCGCTCTCGTGCCAATTCC
271    S  G  L  S  K  W  P  G  S  P  T  T  R  S  T  E  D  L  D  V  W  T  D  F  R  S  R  A  N  S
901   AATGCCAGCACTATAAGTGGTCTGGCAAACCCAGAGTTAGACGAGGTGCCCGACGATGAGCCCCCCCTCTCGCCCATGTTTTACTCCAGT
301    N  A  S  T  I  S  G  L  A  N  P  E  L  D  E  V  P  D  D  E  P  P  L  S  P  M  F  Y  S  S
991   CCTGGTAAAGCTTTGTCTCCTGGCAAAACCACAACCAGTGGCAAGGCTGCACCGGCCGAGCTGCCGCGCCTTGCGGACTTGACGGGTACG
331    P  G  K  A  L  S  P  G  K  T  T  T  S  G  K  A  A  P  A  E  L  P  R  L  A  D  L  T  G  T
1081  ATGAATCTGAATGATGGACTCACAGATGACCTAATGGATGAGTTGCTTGTCAACATCAACCTGGTGCCAACCAGTTCCAGCCAGTCCTCT
361    M  N  L  N  D  G  L  T  D  D  L  M  D  E  L  L  V  N  I  N  L  V  P  T  S  S  S  Q  S  S
1171  CCAAACGATTCATCAGGGTTCACTTTTGGATCCAAACCCAACGGGCTTGTCTCAACTTCCGCCATCTCTTCTTCATCCTCTAATTCTTGT
391    P  N  D  S  S  G  F  T  F  G  S  K  P  N  G  L  V  S  T  S  A  I  S  S  S  S  S  N  S  C
1261  CTCTCTTCTAATGGCAATGGCTTCAGTAACTCCATTTTTGGGCCCCACACGACGGTTTCTCTTCATCCATCCCCAATGCAGACCATCCAG
421    L  S  S  N  G  N  G  F  S  N  S  I  F  G  P  H  T  T  V  S  L  H  P  S  P  M  Q  T  I  Q
1351  GAGAACAAGCAGACCTCCTTCTCCAGCATCTTGTCTAGCTTTGGCAGCCAGACTCTGCAAGATTTGCTGAACTCTGACAGTCACAGCCAC
451    E  N  K  Q  T  S  F  S  S  I  L  S  S  F  G  S  Q  T  L  Q  D  L  L  N  S  D  S  H  S  H
1441  AGTGACGTCATGATGACTCAGTCTGACCCGCTGATGTCACAGGCCAGTGCCGCTGCGGTCATGTCCCAGAATTCCCGACGTGGCCTGATG
481    S  D  V  M  M  T  Q  S  D  P  L  M  S  Q  A  S  A  A  A  V  M  S  Q  N  S  R  R  G  L  M
1531  CTTCGGGATGATCCCGTGATGACTTTTGGAACAACCAGAGGACTTCAGAGCAGCCAAAGGGAGATGCTCCAGAGTAACAACCACAACCAG
511    L  R  D  D  P  V  M  T  F  G  T  T  R  G  L  Q  S  S  Q  R  E  M  L  Q  S  N  N  H  N  Q
1621  AACTCCCTGAGACCTTTGAATGGAGGCCTCAATCTGGCTAATGAGGCCAACAATCTGGCTAATGTTAAGCAGCAGCTCTTGCTGTCACCC
541    N  S  L  R  P  L  N  G  G  L  N  L  A  N  E  A  N  N  L  A  N  V  K  Q  Q  L  L  L  S  P
1711  TTGGGAGGAAACGGCTCGTCCTCTATGCAGATTGACACCTCCATCTTCCTCAATGGAACTGTTAGCAACAATGGAGGCATCTGTCAGGAC
571    L  G  G  N  G  S  S  S  M  Q  I  D  T  S  I  F  L  N  G  T  V  S  N  N  G  G  I  C  Q  D
1801  CGATTCCCACAAGATCTGGACTTGGACATGTTCAGTGGTAGCCTGGAGTGTGACATGGACTCCATCATCAGGACTGAGCTGATGGACGCT
601    R  F  P  Q  D  L  D  L  D  M  F  S  G  S  L  E  C  D  M  D  S  I  I  R  T  E  L  M  D  A
1891  GATGGCCTGGACTTTAACTTTGACTCTATGTCCAACATGAATGGAGTCAGCAACTTCACTAGCACCAATCAGACCTCTCAGAGCTGGGTA
[illegible]
```

Csfoxo4

```
1     ATGGATGAGTCGTCGGTGCCGCCGATTGACCCAGATTTCGAACCACAGAGCAGACCCCGCTCCTGCACATGGCCCCTGCCCAGGCCCGAC
1      M  D  E  S  S  V  P  P  I  D  P  D  F  E  P  Q  S  R  P  R  S  C  T  W  P  L  P  R  P  D
91    ATCTCTACTGTCAAAACGGAGTCCGCGGACGGAGCCGAGTCGGCCGCCGGTACTCCTCCCGCGGACGAGGACAAGGCCGAGCCGCAGCCA
31     I  S  T  V  K  T  E  S  A  D  G  A  E  S  A  A  G  T  P  P  A  D  E  D  K  A  E  P  Q  P
181   ATCACTTCCGAGCCGGAGAAGGCGGTGACGGCCGCAGCCGAGGGCGGAATCGTCGCTGGAGTGGGCGTACCCGGTTCCACTCCACGCAAA
61     I  T  S  E  P  E  K  A  V  T  A  A  A  E  G  G  I  V  A  G  V  G  V  P  G  S  T  P  R  K
271   GGCTCGTCCCGCCGCAACGCGTGGGGAAACCAGAGCTATGCAGACCTGATCTGTCAGGCCATAGAGGAATCACCTGATAAAAGACTGACC
91     G  S  S  R  R  N  A  W  G  N  Q  S  Y  A  D  L  I  C  Q  A  I  E  E  S  P  D  K  R  L  T
361   CTGGCTCAGATCTACGAGTGGATGGTGAAGACGGTGCCTTACTTCAGAGACAAAGGAGACAGCAACAGCTCAGCAGGCTGGAAGAACTCC
121    L  A  Q  I  Y  E  W  M  V  K  T  V  P  Y  F  R  D  K  G  D  S  N  S  S  A  G  W  K  N  S
451   ATTCGCCACAATTTATCACTCCACAACAAGTTTTTGAGGGTCCACAATGAGTCCACAGGAAAGAGCTCCTGGTGGATGCTCAACCCAGAA
151    I  R  H  N  L  S  L  H  N  K  F  L  R  V  H  N  E  S  T  G  K  S  S  W  W  M  L  N  P  E
541   GGAGGCAAAACTGGGAAAGCACCTCGACGAAGGGCAGCTTCCATGGACAACAGCAGTAAACTGTTAAAGAGTCGGATGAGAGCAAAGCAA
181    G  G  K  T  G  K  A  P  R  R  R  A  A  S  M  D  N  S  S  K  L  L  K  S  R  M  R  A  K  Q
631   ACAAAGAAACAGGCGGGAGTGGGCAGTGCTGGAGGAGCACTGCAGGGTGATGGGGGCACGGGGTCAGCTGGTGCAGACAGCCCTAACTCA
211    T  K  K  Q  A  G  V  G  S  A  G  G  A  L  Q  G  D  G  G  T  G  S  A  G  A  D  S  P  N  S
721   TCTCAACAGTTTCCTAAATGGGGAGTCAACAGCAGTAGCCCCTCATCTCGCGGCAGCCTGGATGACAGTGACATGTGGACCACGTTCCGT
241    S  Q  Q  F  P  K  W  G  V  N  S  S  S  P  S  S  R  G  S  L  D  D  S  D  M  W  T  T  F  R
811   CCACGCACCAGCTCCAATGCCAGCACTCTTAGCGGACGTCTGTCCCCTATTGCTCCTGGGCAGGAGGAAGATGACAGCCTGCCTGAAGAC
271    P  R  T  S  S  N  A  S  T  L  S  G  R  L  S  P  I  A  P  G  Q  E  E  D  D  S  L  P  E  D
901   GGGTTGCTGGGAAGATACGCTGCTAGGAGCCTGAGCCCAAGCCTTGCTGAAACTCTCATGGAGGAACTGGATCTGATCGATGGTCTCACA
301    G  L  L  G  R  Y  A  A  R  S  L  S  P  S  L  A  E  T  L  M  E  E  L  D  L  I  D  G  L  T
991   TTGATGACTGGGCAGCAGGGAGGGGCCAGTCCCAGCACAGCTCCACCAGCACCCCCCACCCCACTGCCTTCAGCGTCCACCTTACTACCC
331    L  M  T  G  Q  Q  G  G  A  S  P  S  T  A  P  P  A  P  P  T  P  L  P  S  A  S  T  L  L  P
1081  CGGGGAACCAGCTTCTCCACCTTTCATCAGATGCAAACCTCCAACCTCAAACAGCAGCAGTCCAGCCACACTGCAGCCCAGGCTTCTATC
361    R  G  T  S  F  S  T  F  H  Q  M  Q  T  S  N  L  K  Q  Q  Q  S  S  H  T  A  A  Q  A  S  I
1171  CCCCAATGTGGGCCCAGTGGCAAAGACCCACCCTCCTTCAGTAACTCCCTCTTCAACCCCATGTCCAACTCTGGGTCTCATGGAGCAGT
391    P  Q  C  G  P  S  G  K  D  P  P  S  F  S  N  S  L  F  N  P  M  S  N  S  G  S  H  G  S  S
1261  ACCTACAACACCCATGTGCCCTCATCTCTGGAGGCGCTGCTCACCTCCGACTCCCCTCCTCCCACTGATGTTCTGATGGCGCAGGTGAAC
421    T  Y  N  T  H  V  P  S  S  L  E  A  L  L  T  S  D  S  P  P  P  T  D  V  L  M  A  Q  V  N
1351  CCCATTTTGCCCAGTCCTGGAGGAATGGGGCTAATGGGTCTGAATTCATCTGTGGTGGGTGTGAGGTCAAAACCTAATCAACTTCTGTTA
451    P  I  L  P  S  P  G  G  M  G  L  M  G  L  N  S  S  V  V  G  V  R  S  K  P  N  Q  L  L  L
1441  GGAAAAGGACCAGAGCCAAATGCAGTTGCCCCGATGGCACTGCAGGCCCAGATGCAGCAGCAGCATCACCACCACCATCTTCACAACCAA
481    G  K  G  P  E  P  N  A  V  A  P  M  A  L  Q  A  Q  M  Q  Q  Q  H  H  H  H  H  L  H  N  Q
1531  CACCAGCAGCAGCAGCAGCAGCAGCAAACACAACAACAACAACAGCAGCAGCAGCACCAGTATCACACTCAGATGGGGTTAGGGATGATT
511    H  Q  Q  Q  Q  Q  Q  Q  Q  T  Q  Q  Q  Q  Q  Q  Q  Q  H  Q  Y  H  T  Q  M  G  L  G  M  I
1621  CTCTCAGGTATGTCTCAGGACCCCTCACAACTCTCAGTGCTCAAAGCCCATCATGCCACAGTGCCAGCCATGGGCTCTCTCCACGGAGGG
541    L  S  G  M  S  Q  D  P  S  Q  L  S  V  L  K  A  H  H  A  T  V  P  A  M  G  S  L  H  G  G
1711  CCAGGTCTGAATCTGCAGTCCATGGGTCAGTTTGGAGCACCCACTTGCTTTTCTGCAGGTCAGGACCGTCTGCCCACTGACTTGGATATA
571    P  G  L  N  L  Q  S  M  G  Q  F  G  A  P  T  C  F  S  A  G  Q  D  R  L  P  T  D  L  D  I
1801  GATATGTTCACCGAAAATCTGGATTGTGACGTGGACGACATCATCAACAGTGACCTCATGGATGGAGATGGCCTCGACTTCAACTTTGAC
601    D  M  F  T  E  N  L  D  C  D  V  D  D  I  I  N  S  D  L  M  D  G  D  G  L  D  F  N  F  D
1891  CCCGTTCTGCCTGGCACCCAAGGCTATGCTGGCCCAGCCACCTCACAGGGCTCAACACACAACTGGGTCCCCAGCTAA
631    P  V  L  P  G  T  Q  G  Y  A  G  P  A  T  S  Q  G  S  T  H  N  W  V  P  S  *
```

Csfoxo6-like

```
1       ATGTTGATGATGGAGGACGACGAACTGGACGCTCATCAAGTAGATTCGGATTTCGAGCCGCAGAGCCGGCCTCGCTCCTGCACATGGCCG
1        M  L  M  M  E  D  D  E  L  D  A  H  Q  V  D  S  D  F  E  P  Q  S  R  P  R  S  C  T  W  P
91      CTGCCTTGCCCGGAGGATTTTCCCGGTGCACACGAGGTCAGCGGAGGTCTTCCAGTCACCAGCATAAAGGTGGAACCGGAGGACATCCCG
31       L  P  C  P  E  D  F  P  G  A  H  E  V  S  G  G  L  P  V  T  S  I  K  V  E  P  E  D  I  P
181     GCTTGCAGAGCGGGACTCGCGGTGGGCGGAACGCCGGCAGAGCTGAAGCACCCAGCCGGCGCCCCTGGCGCAGCCCTGGACGCCAACGGA
61       A  C  R  A  G  L  A  V  G  G  T  P  A  E  L  K  H  P  A  G  A  P  G  A  A  L  D  A  N  G
271     CCGCTGCGCAAAGCCAAGTCGTCGCGGCGGAACGCGTGGGGGAACCTGTCCTACGCGGATCTCATTACCCGCGCCATTGAGAGCACACCG
91       P  L  R  K  A  K  S  S  R  R  N  A  W  G  N  L  S  Y  A  D  L  I  T  R  A  I  E  S  T  P
361     GAGAAAAGACTGACGCTGTCACAGATCTACGACTGGATGGTCCGGTACGTGCCCTACTTCAAGGATAAGGGCGACAGCAACAGCTCAGCG
121      E  K  R  L  T  L  S  Q  I  Y  D  W  M  V  R  Y  V  P  Y  F  K  D  K  G  D  S  N  S  S  A
451     GGCTGGAAGAACTCCATCCGGCACAACCTCTCCCTCCACACACGCTTCATCCGGGTGCAGAACGAGGGAACTGGCAAGAGTTCCTGGTGG
151      G  W  K  N  S  I  R  H  N  L  S  L  H  T  R  F  I  R  V  Q  N  E  G  T  G  K  S  S  W  W
541     ATGTTGAATCCAGACGGAGGGAAGATGGGCAAGGCTCCGCGCCGGCGAGCGGTCTCCATGGACAACAGCACTAAGTACTTAAAAAGCAAA
181      M  L  N  P  D  G  G  K  M  G  K  A  P  R  R  R  A  V  S  M  D  N  S  T  K  Y  L  K  S  K
631     GGCCGCATTAGAGGCAAGAGGGTTGGACGTCCTGGAATAGGAGCGGGGTCTGCCGTGACGGGACTCCAGGCCTCCCCGGACCACGGCAGT
211      G  R  I  R  G  K  R  V  G  R  P  G  I  G  A  G  S  A  V  T  G  L  Q  A  S  P  D  H  G  S
721     CCACCACAGAAAACCCTGCCGGGGTTCGCCGCAGCGACCGGGACGGATGGAGAGTTCGACGCCTGGACGGAGCTTCATTCCAGAGCCAGT
241      P  P  Q  K  T  L  P  G  F  A  A  A  T  G  T  D  G  E  F  D  A  W  T  E  L  H  S  R  A  S
811     TCTTCGGCCTCCACCCTCAGCGGGCGTCTGTCACCGATCCTGGCTGAGGAAGAGGCGGAAGAAGCTGAGGTGGGCGGTCTGTCCAGCTCC
271      S  S  A  S  T  L  S  G  R  L  S  P  I  L  A  E  E  E  A  E  E  A  E  V  G  G  L  S  S  S
901     ACATCCCCCCACCTCTACCCGTCGCCAACCAGCACCAGCTCTCCATCAGTGGGGTCTGGGAGCCACTGCCCCCCCCTCCAGCAGCTGCCT
301      T  S  P  H  L  Y  P  S  P  T  S  T  S  S  P  S  V  G  S  G  S  H  C  P  P  L  Q  Q  L  P
991     CAGCTGGCTAACATGGCAGGTGCTATCAGTCTGGATGAGCAGCTGACAGAGGACAGTTATCATCCGCACCGTCAGCAGCAGCAGCAGCAG
331      Q  L  A  N  M  A  G  A  I  S  L  D  E  Q  L  T  E  D  S  Y  H  P  H  R  Q  Q  Q  Q  Q  Q
1081    CAGCAGCAGCAGCAGCAGCACGGCAGACACAAGCACCAAACGCCCGTCTACCACTACAGCTCTGGAATCAAAGGCCAAAGCGGTTACGGC
361      Q  Q  Q  Q  Q  Q  H  G  R  H  K  H  Q  T  P  V  Y  H  Y  S  S  G  I  K  G  Q  S  G  Y  G
1171    CCCGGTGTCTACGGGGCCTCGGCCATGGGGATGCTGCATCACCACTCACCCCATATGCAGACGATTCAAGAGAACAAGGCGGCCAGTTTC
391      P  G  V  Y  G  A  S  A  M  G  M  L  H  H  H  S  P  H  M  Q  T  I  Q  E  N  K  A  A  S  F
1261    TCTGGAACCATGCGGGCGTACTCCGGTGCCAACGCCCTGCAGACGCTGCTCACCGGGGGTCCAGCTGGGCAGCAGTACTGCTCCAAGGAC
421      S  G  T  M  R  A  Y  S  G  A  N  A  L  Q  T  L  L  T  G  G  P  A  G  Q  Q  Y  C  S  K  D
1351    ATGCTGCTTGGACAGGAGCGAGAAAGCCATCCCATGATCGGACAGCCCAGCAACGGGGTGGGCCCCAACCATCACAGCCACCATCACGGT
451      M  L  L  G  Q  E  R  E  S  H  P  M  I  G  Q  P  S  N  G  V  G  P  N  H  H  S  H  H  H  G
1441    CACCACAACGGCCACAGCGAACCACACGGTCACCGCACAGCCCACAGCCATAACAGAACTCCCAGCCATCACAATCACAACGGTGTCGCC
481      H  H  N  G  H  S  E  P  H  G  H  R  T  A  H  S  H  N  R  T  P  S  H  H  N  H  N  G  V  A
1531    AACCGCAACCACAGCTCACCTCACGGCCACAGTCACAACACGCACAACCTCAACCACAGCCATAACCACACACCACCCAGGGCAGCGGTG
511      N  R  N  H  S  S  P  H  G  H  S  H  N  T  H  N  L  N  H  S  H  N  H  T  P  P  R  A  A  V
1621    CTAGCCCCCAGCGGCAACAACAATCACCTGCAGACCTACAACCACAAAACTCCCTACTTGTACAGCCCCCCCTCACACGCCCACCTCCCG
541      L  A  P  S  G  N  N  N  H  L  Q  T  Y  N  H  K  T  P  Y  L  Y  S  P  P  S  H  A  H  L  P
1711    GCCTCCACCACCCTCCCTCCCAACCCGGCGGGTATGCTCGGCATGCCTCAGGACTTCTGCCACGTTGCCACCGCCCCCCACCCTCACCCT
571      A  S  T  T  L  P  P  N  P  A  G  M  L  G  M  P  Q  D  F  C  H  V  A  T  A  P  H  P  H  P
1801    CGTCACAGCCACTATCCCGACCCGCAGCATCAGGGCGTGACTAGTGGATCGTACCCGTACGGCCCGGGGGTGGGCAACGGTACCACGGGT
601      R  H  S  H  Y  P  D  P  Q  H  Q  G  V  T  S  G  S  Y  P  Y  G  P  G  V  G  N  G  T  T  G
1891    AGCAACTACCACAGCTATCACCAACCGCACGCCCACGAGAGACTACCGGCCGATTTGGACATTGACATGTTCCACGGCAGCTTGGACTGC
631      S  N  Y  H  S  Y  H  Q  P  H  A  H  E  R  L  P  A  D  L  D  I  D  M  F  H  G  S  L  D  C
1981    GACGTGGAGTCCATTCTCCTCCATGACATTATGGACTCTGGGGAGGAGATGGACTTTAACTTTGACTGCTCCCTCGCTCAGGGTGTGGGC
661      D  V  E  S  I  L  L  H  D  I  M  D  S  G  E  E  M  D  F  N  F  D  C  S  L  A  Q  G  V  G
2071    ATCGGCATGGGGATGGGCATGGGGGTCACCATGGGCATGGGGATGGGAATGAGCAGCCTGGCCAGTCCTCAGCAGGCCCACAGTAATCAG
691      I  G  M  G  M  G  M  G  V  T  M  G  M  G  M  G  M  S  S  L  A  S  P  Q  Q  A  H  S  N  Q
2161    AGCTGGGTGCCAGGCTGA
721      S  W  V  P  G  *
```

Csfoxo1a-like

```
1       ATGGCTGAGGTCCCGACACAGACGGCTGACATCGACCCGGACTTTGAGCCTCTTTCCCGCCCGCGGTCGTGCACTTGGCCCCTGCCGCGG
1        M  A  E  V  P  T  Q  T  A  D  I  D  P  D  F  E  P  L  S  R  P  R  S  C  T  W  P  L  P  R
91      CCGGAGCTCCTGGACCCCGCCGGCTCTAACACGTCCTCCCCGGCGCCGTCGGTGCAGCAGGAGCTCGGAGGAAACGCCGAGTTCATCAGT
31       P  E  L  L  D  P  A  G  S  N  T  S  S  P  A  P  S  V  Q  Q  E  L  G  G  N  A  E  F  I  S
181     AACCTGGGCTTGTTGGAGGAGGACTACGAGGAGTATGAGGAGCAGAAACAACCCGTGCATCTTCATCACAACCATCGCCACCATCATCAC
61       N  L  G  L  L  E  E  D  Y  E  E  Y  E  E  Q  K  Q  P  V  H  L  H  H  N  H  R  H  H  H  H
271     CATCACCTCCAGCAGCAGCAGCAGGTCCCTGGTCTGCAGCAGCAGCAGCAGGCGCCGCCGGCCGCCGGCACAACGCGCCCCGCCTCTCCG
91       H  H  L  Q  Q  Q  Q  Q  V  P  G  L  Q  Q  Q  Q  Q  A  P  P  A  A  G  T  T  R  P  A  S  P
361     CGCTGTGGGGTGGCCGCCAGAGGGAAGGCCGCCCCCGGCCCCTCATCCCGCCGTAACGCCTGGGGGAACATGTCGTACGCAGACCTGATC
121      R  C  G  V  A  A  R  G  K  A  A  P  G  P  S  S  R  R  N  A  W  G  N  M  S  Y  A  D  L  I
451     ACCAAAGCCATCGAGAGCTCCCCGGAGAAGAGGCTGACTCTGTCCCAGATCTACGACTGGATGGTGAAGAGCGTGCCGTACTTCAAGGAT
151      T  K  A  I  E  S  S  P  E  K  R  L  T  L  S  Q  I  Y  D  W  M  V  K  S  V  P  Y  F  K  D
541     AAAGGCGACACCAACAGCTCTGCAGGCTGGAAGAACTCCATCAGACACAACCTGTCCCTGCACAGCCGCTTCATTCGCATTCAGAATGAA
181      K  G  D  T  N  S  S  A  G  W  K  N  S  I  R  H  N  L  S  L  H  S  R  F  I  R  I  Q  N  E
631     GGAACGGGAAAAAGTTCCTGGTGGATGTTGAACCCTGAAGGAGGGAAGAACGGAAAGTCCCCGCGACGCAGAGCCGCCTCCATGGACAAC
211      G  T  G  K  S  S  W  W  M  L  N  P  E  G  G  K  N  G  K  S  P  R  R  R  A  A  S  M  D  N
721     AACAGCAAACTGGCCAAGAGCAGAGGAAGAGCCACGAAGAAGAAGATGGCCCTGCAGGGGGAGGCAGAGGGAGGAGGGGGGAGTCCTGGT
241      N  S  K  L  A  K  S  R  G  R  A  T  K  K  K  M  A  L  Q  G  E  A  E  G  G  G  G  S  P  G
811     TCCCTCTACTCCAAGTGGCTGGGGAGTCCAAACTCTCTCAGTAACGAGGACTTTGAGTCCTGGAACTCTTTTAGGACACGCACCAGCTCG
271      S  L  Y  S  K  W  L  G  S  P  N  S  L  S  N  E  D  F  E  S  W  N  S  F  R  T  R  T  S  S
901     GACGCCAGCACTCTCAGCGGTCACCGTTCACCCTTCCCTCCGGAGCAGGATGACCTGGGGGAGCCAGAAGGTCACATGATGTACCCCGGG
301      D  A  S  T  L  S  G  H  R  S  P  F  P  P  E  Q  D  D  L  G  E  P  E  G  H  M  M  Y  P  G
991     GCAGCGGGGGCCAAGATAACCTCCACCCTGCCCAGTCTGTCTGAGATGGGCGGGTCTCTGGGCCAGGATGAGTCGGAGATCGTCATGGGA
331      A  A  G  A  K  I  T  S  T  L  P  S  L  S  E  M  G  G  S  L  G  Q  D  E  S  E  I  V  M  G
1081    ACTCTGTTGGATAATCTGGACCTGCTGTCGCCCAAGAACCCCAAGGTGGCGTCTGACTCCCCCAGCATGCTTCAGAGCGCCCCCTACAGC
361      T  L  L  D  N  L  D  L  L  S  P  K  N  P  K  V  A  S  D  S  P  S  M  L  Q  S  A  P  Y  S
1171    ACAGCCACCTTACCCCCACTGTCGCAGCAGGATTATCGTAAATGCATGTACGGCCAAGTGGGCATGGAGCCCCGCCCCCCTGCCCCCATG
391      T  A  T  L  P  P  L  S  Q  Q  D  Y  R  K  C  M  Y  G  Q  V  G  M  E  P  R  P  P  A  P  M
1261    CAGACACTTCAAGAAACCAAGGTGGGCTTCGGGGTCTACGAAAACCAATATGTCTGCCCTCAGGGACTCCTCAAGGAGCTGCTGAGCGCG
421      Q  T  L  Q  E  T  K  V  G  F  G  V  Y  E  N  Q  Y  V  C  P  Q  G  L  L  K  E  L  L  S  A
1351    GAGGCCGATGGCAGCAGGGACATGATGCCCTGCAGGAACGCACTGGTGCCTCAGGTGGGGAGGGGAGGGTGTCTGATGCCCACCTACAGC
451      E  A  D  G  S  R  D  M  M  P  C  R  N  A  L  V  P  Q  V  G  R  G  G  C  L  M  P  T  Y  S
1441    AGGCAGAGTCACGTCGGCGGGGGGGTGAAGATGATGAACCTCCCACACCCACTCACTGTCCTCAACCAAGGACCTTCGACCTCCAGGGAC
481      R  Q  S  H  V  G  G  G  V  K  M  M  N  L  P  H  P  L  T  V  L  N  Q  G  P  S  T  S  R  D
1531    TTGAACAGCTGTAACATGATCCCACTGACGGGTCCTTCAGGGGCCCCTCCTGCAGTGACCGGCCCCAGGACCCGGGGTCAGCCTGCGCAC
511      L  N  S  C  N  M  I  P  L  T  G  P  S  G  A  P  P  A  V  T  G  P  R  T  R  G  Q  P  A  H
1621    ACACACATGAACTACGGCCGGAGCTACAGAGAACTGACGTCCGTCCACGCCCACGGTCACCACCACCAGGAGCGACTGCCCAGTGATCTG
541      T  H  M  N  Y  G  R  S  Y  R  E  L  T  S  V  H  A  H  G  H  H  H  Q  E  R  L  P  S  D  L
1711    GACAACATGTCCATCGAGATGTTTGAATGTGACATGGAGTCCGTCCTCCACGACACCCTCATGGACGGCGGGGCCTTGGACTTCAACTTT
571      D  N  M  S  I  E  M  F  E  C  D  M  E  S  V  L  H  D  T  L  M  D  G  G  A  L  D  F  N  F
1801    GACCCTGCAGCTGCCTCTCACGGGTTTCCCCAAAGGGTAAAAGGCAACTCTCACAGCTGGGTGTCAGGCTAG
601      D  P  A  A  A  S  H  G  F  P  Q  R  V  K  G  N  S  H  S  W  V  S  G  *
```

Supplementary Figure S2 Putative motifs of foxo proteins

	Logo	E-value	Sites	Width	More	Submit/Download
1.	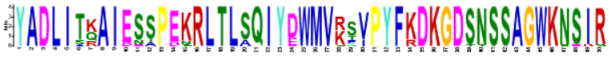	3.1e-1266	27	50		
2.	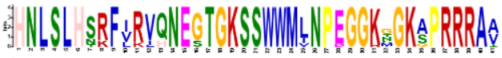	1.4e-957	27	41		
3.	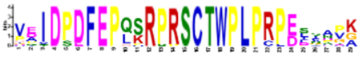	4.6e-522	26	29		
4.	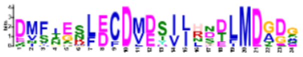	1.9e-341	27	24		
5.	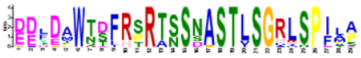	7.3e-449	27	29		
6.	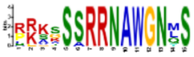	5.1e-232	27	15		
7.	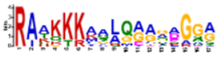	1.6e-137	27	17		
8.	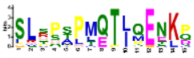	1.3e-142	27	15		
9.	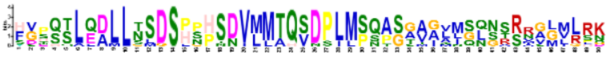	6.6e-208	13	50		
10.	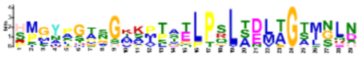	1.1e-145	21	29		
11.	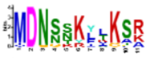	7.1e-123	27	11		
12.	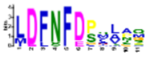	3.2e-111	27	11		

Stopped because requested number of motifs (12) found.

Supplementary Figure S3 The structure and motif analysis of Foxo proteins in three kind of mammals，mouse (Mmfoxo)，humans (Hsfoxo) and Rattus norvegicus (Rnfoxo).

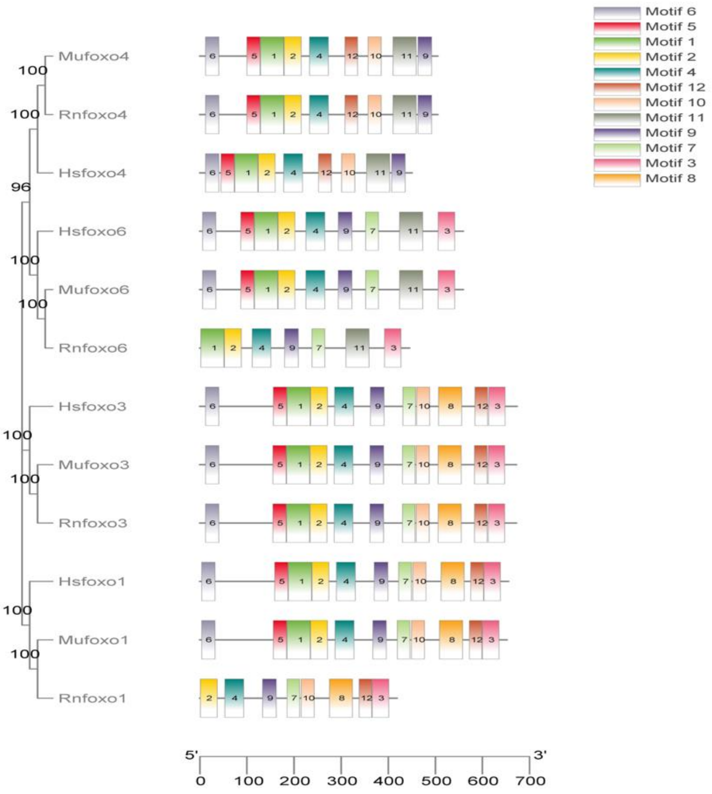

Supplementary Table S1

List of species used in this study

No.	Species name	Accession ID	Source
1	*Cynoglossus semilaevis*	XP_024912472.1<br>XP_008325121.1<br>XP_008309103.1<br>XP_008307478.1<br>XP_008320914.1<br>XP_008331716.2	NCBI
2	*Oryzias latipes*	XP_011489778.1<br>XP_023820018.1<br>XP_011480493.1<br>XP_023815809.1<br>XP_004076562.1<br>XP_023814834.1	NCBI
3	*Scophthalmus maximus*	XP_035474393.1<br>XP_035506031.1<br>XP_035479973.1	NCBI
4	*Danio rerio*	NP_001070725.2<br>NP_571160.1<br>NP_001009988.1<br>NP_001076326.1<br>XP_009289443.1<br>NP_001128604.2<br>XP_690041.2	NCBI
5	*Lepisosteus oculatus*	XP_006631437.1<br>XP_006626029.1<br>XP_015207025.1<br>XP_006628257.1	NCBI
6	*Oreochromis niloticus*	XP_005477409.1<br>XP_005467551.1<br>XP_025766550.1<br>XP_005454675.1<br>XP_003455269.2<br>XP_003450195.1	NCBI
7	*Takifugu rubripes*	XP_029699608.1<br>XP_029703679.1<br>XP_003978591.1<br>XP_003971932.1<br>XP_003969118.2<br>XP_011609846.1	NCBI

8	*Perca fluviatilis*	XP_039641440.1<br>XP_039638602.1<br>XP_039633989.1<br>XP_039668563.1	NCBI
9	*Hippoglossus hippoglossus*	XP_034456251.1<br>XP_034436699.1<br>XP_034432342.1	NCBI
10	*Mus musculus*	NP_062713.2<br>NP_001363896.1<br>NP_061259.1<br>NP_918949.1	NCBI
11	*Homo sapiens*	NP_001446.1<br>NP_002006.2<br>NP_001164402.1<br>NP_001278210.2	NCBI

Supplementary Table S2

Primers and their sequences in this study.

Primer	Sequence (5′-3′)	Information
Csfoxo1a-RT-F	GTCGTCCCCGTCTCCTATGAT	qRT-PCR
Csfoxo1a-RT-R	AAAACTGGGCTTGCTCTCTGG	qRT-PCR
Csfoxo3a-RT-F	GAGGACGAGCCACCAAGAAA	qRT-PCR
Csfoxo3a-RT-R	GCTGAGGGTGCTGGCATTAGA	qRT-PCR
Csfoxo3b-RT-F	TTGCTTGTCAACATCAACCTG	qRT-PCR
Csfoxo3b-RT-R	TGAAGAGAAACCGTCGTGTG	qRT-PCR
Csfoxo4-RT-F	AGACGGTGCCTTACTTCAGAG	qRT-PCR
Csfoxo4-RT-R	GGTGCTTTCCCAGTTTTGC	qRT-PCR
Csfoxo6-like-RT-F	CAGTTATCATCCGCACCGTC	qRT-PCR
Csfoxo6-like-RT-R	GCCTTGTTCTCTTGAATCGTCT	qRT-PCR
Csfoxo1a-like-RT-F	AGAGGAAGAGCCACGAAGAA	qRT-PCR
Csfoxo1a-like-RT-R	GCTGGTGCGTGTCCTAA	qRT-PCR
β-actin-F	TTCCAGCCTTCCTTCCTT	qRT-PCR
β-actin-F	TACCTCCAGACAGCACAG	qRT-PCR
Csfoxo1a-P-F	ATCTGCGATCTAAGTAAGCTTGTGAAGCGTCCCAATA	promoter
Csfoxo1a-P-R	CAGTACCGGAATGCCAAGCTTGACAGCAGACGAGCACT	promoter
Csfoxo3a-P-F	ATCTGCGATCTAAGTAAGCTTGCCTTGTAGGGAGATT	promoter
Csfoxo3a-P-R	CAGTACCGGAATGCCAAGCTTAATCTGCTGCTGTGG	promoter

Csfoxo3b-P-F	ATCTGCGATCTAAGTAAGCTTTTTGATATTGTCCCAACTAG	promoter
Csfoxo3b-P-R	CAGTACCGGAATGCCAAGCTTGTCGGTGTCAATGTGGA	promoter
Csfoxo4-P-F	ATCTGCGATCTAAGTAAGCTTGAGCCCTGACACGAAC	promoter
Csfoxo4-P-R	CAGTACCGGAATGCCAAGCTTAAAGTCAATGGACCACC	promoter
mufoxo1a-C/EBPα-F	AACACGTTTAAAGGACGTTTAGCTTCTTAAGATAAC	binding site mutation
mufoxo1a-C/EBPα-R	GTTATCTTAAGAAGCTAAACGTCCTTTAAACGTGTT	binding site mutation
mufoxo1a-c-Jun-F	ATCTCATTATGAACTGACCTAATTCGTTT	binding site mutation
mufoxo1a- c-Jun -R	AAACGAATTAGGTCAGTTCATAATGAGAT	binding site mutation
mufoxo3a-C/EBPα-F	ATCGCAACATACGTGTTTAGGAAAAAAATGT	binding site mutation
mufoxo3a-C/EBPα-R	ACATTTTTTTCCTAAACACGTATGTTGCGAT	binding site mutation
mufoxo3a-c-Jun-F	TAACTTTGACTCTGAGACACAAAAAATGTCA	binding site mutation
mufoxo3a- c-Jun -R	TGACATTTTTTGTGTCTCAGAGTCAAAGTTA	binding site mutation
mufoxo3b-C/EBPα-F	ACTCGCCCTCTCTGACTGCTGTGAAGTTAA	binding site mutation
mufoxo3b-C/EBPα-R	TTAACTTCACAGCAGTCAGAGAGGGCGAGT	binding site mutation
mufoxo3b-c-Jun-F	CCTCACTGAGCGCTGTGAAGT	binding site mutation
mufoxo3b- c-Jun -R	ACTTCACAGCGCTCAGTGAGG	binding site mutation
mufoxo4-C/EBPα-F	GCAGAGCCAGATCCCTGACAGTGG	binding site mutation
mufoxo4-C/EBPα-R	CCACTGTCAGGGATCTGGCTCTGC	binding site mutation
mufoxo4-c-Jun-F	AGTTATCTGTGTGCATGGTTTAATC	binding site mutation
mufoxo4- c-Jun -R	GATTAAACCATGCACACAGATAACT	binding site mutation
Csfoxo1a-siRNA-F1	CCCAGAUCUAUGACUGGAUTT	siRNA
Csfoxo1a-siRNA-R1	AUCCAGUCAUAGAUCUGGGTT	siRNA
Csfoxo1a-siRNA-F2	GCCAUCUAUGCCACUGCAATT	siRNA
Csfoxo1a-siRNA-R2	UUGCAGUGGCAUAGAUGGCTT	siRNA
Csfoxo1a-siRNA-F3	GCACAACCAGGCUAAGAAUTT	siRNA
Csfoxo1a-siRNA-R3	AUUCUUAGCCUGGUUGUGCTT	siRNA
Csfoxo3a-siRNA-F1	GAGGAAGACAACGGAGUUATT	siRNA
Csfoxo3a-siRNA-R1	UAACUCCGUUGUCUUCCUCTT	siRNA
Csfoxo3a-siRNA-F2	GACCAUUCAGGAGAACAAATT	siRNA
Csfoxo3a-siRNA-R2	UUUGUUCUCCUGAAUGGUCTT	siRNA
Csfoxo3a-siRNA-F3	GACUGUGACAUGGACUCUATT	siRNA
Csfoxo3a-siRNA-R3	UAGAGUCCAUGUCACAGUCTT	siRNA
Csfoxo3b-siRNA-F1	GACAACAGCAACAAGUACATT	siRNA
Csfoxo3b-siRNA-R1	UGUACUUGUUGCUGUUGUCTT	siRNA

Csfoxo3b-siRNA-F2	GAUGACCUAAUGGAUGAGUTT	siRNA
Csfoxo3b-siRNA-R2	ACUCAUCCAUUAGGUCAUCTT	siRNA
Csfoxo3b-siRNA-F3	GGACCGAUUCCCACAAGAUTT	siRNA
Csfoxo3b-siRNA-R3	AUCUUGUGGGAAUCGGUCCTT	siRNA
IGF1-RT-F	GTATCTCCTGTAGCCACACCCTCT	qRT-PCR
IGF1-RT-R	GCCTCTCTCTCCACACACAAACT	qRT-PCR
Fox12-RT-F	GAGAGGAAGGGCAACTACTGGA	qRT-PCR
Fox12-RT-R	TGGTTGGAAGTGCGTGGG	qRT-PCR
nerul3-RT-F	CTGGTGTTTAGCAGCCGTCCT	qRT-PCR
nerul3-RT-R	CCAGAACTCCAGCACTGACCC	qRT-PCR
tesk1-RT-F	GCAGAAACTCTCTCACCCCAACA	qRT-PCR
tesk1-RT-R	CCAGACCAAAGTCCGTCACCA	qRT-PCR
wt1a-RT-F	ACCGCCGTTTCCCCTTAC	qRT-PCR
wt1a-RT-R	GGGCTGGTGGTGATGTGC	qRT-PCR
sox9a-RT-F	AAGAACCACACAGATCAAGACAGA	qRT-PCR
sox9a-RT-R	TAGTCATACTGTGCTCTGGTGATG	qRT-PCR
